# Patient Preferences for Unresectable Hepatocellular Carcinoma Treatments: A Discrete-Choice Experiment

**DOI:** 10.3390/cancers15051470

**Published:** 2023-02-25

**Authors:** Daneng Li, Ruoding Tan, Sairy Hernandez, Norelle Reilly, Cooper Bussberg, Carol Mansfield

**Affiliations:** 1 City of Hope Comprehensive Cancer Center, Duarte, CA 91010, USA; 2 Genentech, Inc., South San Francisco, CA 94080, USA; 3 RTI Health Solutions, Research Triangle Park, NC 27709, USA

**Keywords:** discrete choice, stated preferences, survival, health-related quality of life

## Abstract

**Simple Summary:**

Several treatments are available for patients with advanced hepatocellular carcinoma (HCC), and it is important to understand patients’ treatment priorities and goals regarding such treatment options. In a survey study, we explored 200 patients’ preferences for six different features of HCC treatments: months of additional survival, months of maintained daily function, severity of hand-foot syndrome, severity of high blood pressure, risk of bleeding in the digestive tract, and how the medicine is taken. Of the features included in the survey, it was most important to respondents to avoid moderate-to-severe hand-foot syndrome and moderate-to-severe high blood pressure. Respondents considered 10 additional months of maintaining daily functioning to be as important or more important than 10 additional months of survival. For some patients with HCC, maintaining quality of life and avoiding moderate-to-severe side effects may be as important or more important than a medicine’s survival benefit.

**Abstract:**

Treatments for unresectable hepatocellular carcinoma (HCC) have varying benefit-risk profiles. We elicited 200 US patients’ preferences for attributes associated with various first-line systemic treatments for unresectable HCC in a discrete-choice experiment (DCE) survey. Respondents answered nine DCE questions, each offering a choice between two hypothetical treatment profiles defined by six attributes with varying levels: overall survival (OS), months of maintained daily function, severity of palmar-plantar syndrome, severity of hypertension, risk of digestive-tract bleeding, and mode and frequency of administration. A random-parameters logit model was used to analyze the preference data. Patients regarded an additional 10 months of maintaining daily function without decline to be as important or more important than 10 additional months of OS, on average. Respondents valued avoiding moderate-to-severe palmar-plantar syndrome and hypertension more than extended OS. A respondent would require >10 additional months of OS (the greatest increase presented in the study) on average to offset the increased burden of adverse events. Patients with unresectable HCC prioritize avoiding adverse events that would severely impact their quality of life over mode and frequency of administration or digestive-tract bleeding risk. For some patients with unresectable HCC, maintaining daily functioning is as important or more important than the survival benefit of a treatment.

## 1. Introduction

In 2022, more than 41,000 new cases of liver cancer will be diagnosed and an estimated 30,500 deaths attributable to liver cancer will occur [1]. Hepatocellular carcinoma (HCC), the most common form of liver cancer, is among the leading causes of cancer-related mortality worldwide [2]. Because of the disease’s asymptomatic nature in early stages, most HCC cases are detected in advanced stages, often leading to incurable disease states. Symptoms of advanced HCC include appetite loss, weight loss, gastrointestinal symptoms (such as nausea or vomiting), hepatomegaly or splenomegaly, abdominal pain, ascites, pruritus, and jaundice [3]. Many patients with advanced HCC have concomitant liver disease and are at increased risk for adverse events (AEs) while undergoing treatment for HCC because of baseline hepatic dysfunction or comorbidities.

The cancer immunotherapy (CIT) combination atezolizumab plus bevacizumab has become the new standard-of-care treatment, designated as a National Comprehensive Cancer Network Category 1 preferred regimen for unresectable HCC in the United States (US) [4,5]. Before the introduction of CIT, treatment options were limited to oral tyrosine kinase inhibitors (e.g., sorafenib, lenvatinib) that yield modest survival benefits and are associated with AEs including hypertension, palmar-plantar syndrome, and diarrhea [6]. Relative to sorafenib, atezolizumab plus bevacizumab has resulted in significant improvements in overall survival (OS) and progression-free survival outcomes, as well as prolonged time to deterioration in health-related quality of life (HRQOL) and functioning [7,8]. AEs commonly associated with atezolizumab plus bevacizumab include proteinuria, hypertension, and fatigue [4,7]. Upper gastrointestinal bleeding is a common and life-threatening complication in patients who have cirrhosis and hepatocellular carcinoma, and gastrointestinal bleeding, including fatal bleeding events, is a known adverse reaction to bevacizumab [4,7]. The addition of atezolizumab plus bevacizumab contributes to the multiple treatment options available for patients with unresectable HCC, and with new CIT regimens in the pipeline patients with unresectable HCC will have multiple treatment options to consider.

Unfortunately, patients with unresectable HCC often have poor prognosis and may experience impairments in HRQOL because of underlying liver disease and AEs associated with treatment. As there are many available treatment options that vary in benefit-risk profiles, patients’ preferences in treatment selection are becoming increasingly important. Treatment plans should be individualized to align with patients’ values and preferences, using a shared decision-making approach [9]. Recent research has focused on preferences for treatment attributes associated with systemic therapies and selective internal radiation therapy among patients in four European countries undergoing treatment for HCC [10]. However, additional research is needed to understand what is important to patients in the US when choosing a treatment for advanced HCC in an evolving systemic treatment landscape. In order to address this knowledge gap, we implemented a discrete-choice experiment (DCE) approach to elicit US patients’ benefit-risk preferences for attributes associated with first-line systemic treatments approved in the US for the treatment of unresectable HCC. DCEs have been widely used to measure the preferences of patients and have been applied broadly in healthcare decision making [11]. DCE methods are based on the principles that (1) individuals evaluate treatments on the basis of the treatment attributes and the levels of those attributes, and (2) an individual’s choice of treatment is dependent on the importance of each treatment attribute relative to the other attributes.

## 2. Materials and Methods

### 2.1. Study Design

**Figure 1:**
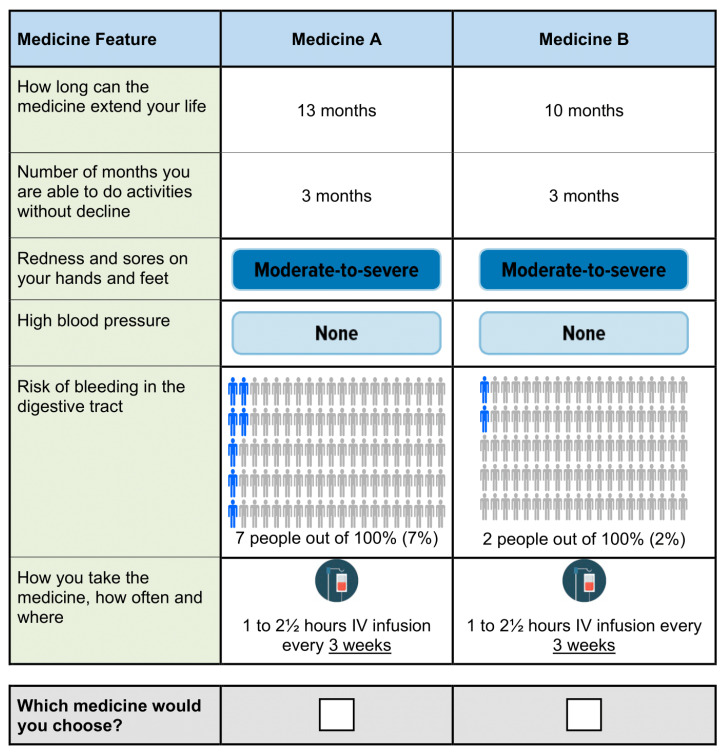
Example discrete-choice experiment question.

A cross-sectional, web-based DCE survey was developed and administered. In this study, patients with unresectable HCC were asked, in a series of experimentally designed questions, to choose between pairs of hypothetical treatments, each defined by a series of attributes and levels, which were identified to represent features of currently available systemic therapies for HCC (see Figure 1). By analyzing which combinations of attributes and levels respondents chose, we were able to estimate the relative importance of different attributes associated with various HCC treatments and understand the tradeoffs respondents are willing to make among attributes.

We conducted qualitative interviews with 16 patients with unresectable HCC to learn about treatment attributes of importance to patients and thereby inform the development of the survey instrument. Candidate attributes were developed based on the patient input, input from clinical experts, and a targeted literature review of data on existing HCC treatments [6,7,12,13,14,15,16,17].

Table 1 summarizes the final set of 6 attributes included in the survey: OS, number of months to maintain daily function, severity of palmar-plantar syndrome, severity of hypertension, risk of bleeding in the digestive tract, and mode and frequency of administration. For each attribute, a range of levels was selected based on the available data and known characteristics of the treatments. The range of levels of each attribute was selected to span the clinically relevant range of outcomes observed or expected in clinical trials or clinical practice. As presented in Table 1, mild palmar-plantar syndrome corresponded with Grade 1, while moderate-to-severe corresponded with Grade 2 or higher [18]. The description of moderate-to-severe hypertension described the side effects and serious risks associated with elevated blood pressure.

A draft version of the survey instrument was pretested in qualitative, semi-structured cognitive debriefing interviews with a convenience sample of 12 US patients with unresectable HCC. The pretests evaluated participants’ comprehension of the DCE survey instrument, and the findings were used to refine the survey and inform the data analysis approach and interpretation of the results. In the final survey, the identified attributes and levels were combined using an experimental design to define the series of hypothetical treatment profiles for unresectable HCC treatments between which respondents were asked to choose. The experimental design was developed using SAS statistical software version 9.4, following good research practice guidelines [19]. The full fractional experimental design included a total of 72 DCE questions that were used to create 8 blocks, each with 9 DCE questions. Respondents were randomly assigned to 1 block of 9 questions, and within each block the questions were randomly ordered to avoid ordering effects. The final survey was programmed and administered online. The RTI International institutional review board reviewed the study protocol and deemed the study exempt from full review. All respondents provided online informed consent.

### 2.2. Study Population

The international healthcare research organization Global Perspectives recruited patients with HCC in the US and confirmed their eligibility to participate in the survey. Eligible respondents self-reported having received a physician diagnosis of unresectable HCC (i.e., HCC that was not eligible for resection or transplantation), were US residents, were aged 18 years or older, and were able to read and understand English to provide informed consent.

### 2.3. Data Analysis

#### 2.3.1. Preference Weights

A random-parameters logit (RPL) model was used to analyze the DCE data. The RPL model related the choices respondents made to the differences in the attribute levels across the alternatives in each choice question [20]. Statistical analysis of the DCE data followed good research practice guidelines published by ISPOR [20] and was performed in STATA 16 and 17. The resulting log-odds parameter estimates from the RPL model can be interpreted as preference weights indicating the strength of preferences for each attribute level relative to the other attributes and levels included in the experimental design.

#### 2.3.2. Conditional Relative Importance

The difference between the RPL preference weights for the most and least preferred levels within each attribute can be interpreted as an estimate of the overall importance patients placed on one attribute relative to the other attributes, conditional on the selected levels and attributes in the design. We summed across all attribute-specific differences and scaled the sum to 100; the conditional importance of each attribute is a percentage of this total. We also performed a subgroup analysis to assess whether relative attribute importance differs by key patient characteristics (see Appendix C, Table A2).

#### 2.3.3. Minimum Overall Survival Required to Offset Changes in Other Treatment Attributes

To explore respondents’ willingness to trade off between specific attributes and levels in the study, the preference weights were used to calculate the minimum acceptable benefit (MAB), or the minimum acceptable gain in months of OS, that the average respondent would require to offset an increase in the risk of treatment-related AEs or a worsening in the level of another treatment attribute. MABs provide another way to quantify the relative importance of changes from one level of an attribute to another and can be understood as the minimum additional months of OS patients need to balance deterioration in other attributes. When the 95% confidence interval around a mean MAB includes 0, the mean MAB is not statistically different from 0.

#### 2.3.4. Simulation

Finally, we conducted a simulation exercise to explore the likelihood that a respondent would choose a particular treatment when presented with the option of 2 treatment profiles similar to real-world therapies. In this exercise, which was conducted in 3 distinct scenarios, we used the preference weight estimates to predict the probability that one treatment would be selected over another in each given scenario.

## 3. Results

### 3.1. Respondent Characteristics

Of 211 potential respondents who were approached to participate, a total of 200 respondents (94.8%) with self-reported unresectable HCC in the US met the eligibility criteria, completed the screening questions, and consented to complete the survey. Respondents had a mean age of 59 years, and 55.5% were male; 28.5% were Black or African American, 21.0% were White, 14.5% were Hispanic or Latino, and 41.0% were an unknown/unreported race (Table 2). Most respondents had received an HCC diagnosis fewer than 5 years ago (93.0%) and were receiving treatment for HCC at the time of the survey (85.5%); 23.0% reported needing help taking care of themselves.

### 3.2. Preference Weights and Conditional Relative Importance

**Figure 2:**
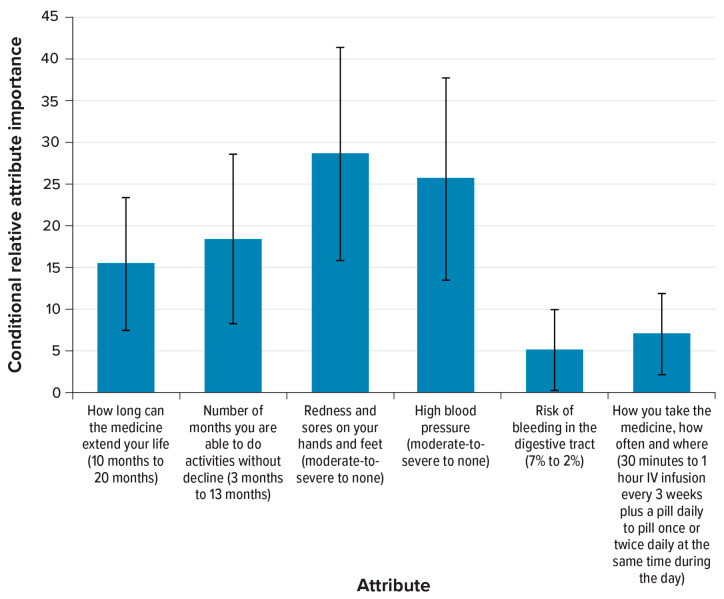
Conditional relative importance of attributes for respondents (*N* = 200). IV = intravenous. Note: The conditional relative importance is the difference between the most-preferred and least-preferred preference weights. These differences are summed across attributes, and this sum is scaled to 100. The conditional importance of each attribute is a percentage of this sum total. The vertical bars surrounding each relative importance weight estimate indicate the 95% confidence interval around the point estimate, computed by the delta method.

**Figure A1:**
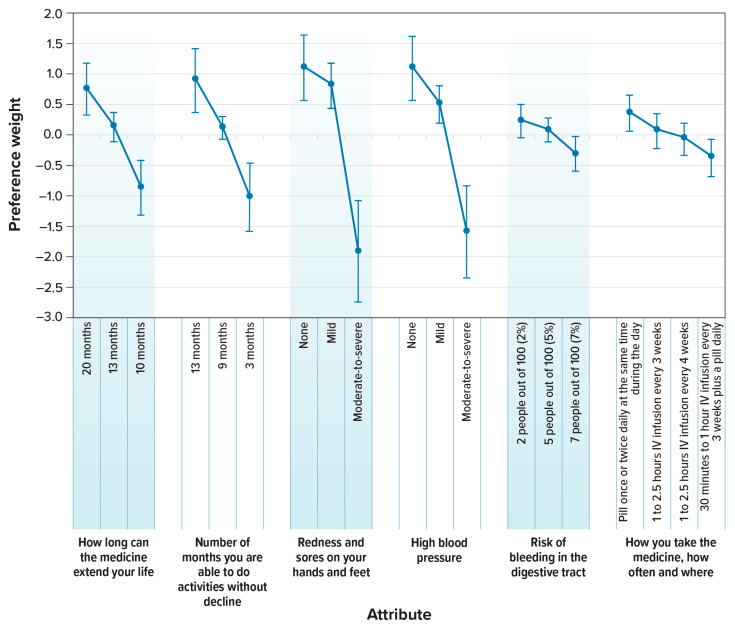
Attribute Preference Weights for Respondents (*N* = 200). IV = intravenous. Note: The change in utility associated with a change from 1 level of an attribute to another for each attribute is represented by the difference between the preference weights between the levels. Larger differences between preference weights indicate that respondents viewed the change as having a relatively greater effect on overall utility. The parameter estimates are the preference weights corresponding to the effects-coded attribute levels and the dummy-coded alternative specific constant. The effects-coded variables are categorical variables ranging from −1 to 1. The preference weights corresponding to the effects-coded variables are log odds, which are distributed symmetrically around 0. The vertical bars surrounding each mean preference weight denote the 95% confidence interval of the point estimate (computed by the delta method for the level omitted in estimation for model specification).

The preference weight estimates reveal that respondents preferred treatments that extend life longer, maintain daily function longer, cause less-severe palmar-plantar syndrome and less-severe hypertension, have lower risk of bleeding in the digestive tract, and can be taken by pill once or twice daily (see Figure A1, Appendix B). Figure 2 shows the overall relative importance for one attribute relative to the others included in the survey. Respondents placed the most importance on avoiding moderate-to-severe palmar-plantar syndrome and avoiding moderate-to-severe hypertension. However, the preference weight estimates for mild levels of palmar-plantar syndrome and hypertension were not significantly different from no palmar-plantar syndrome and no hypertension, implying that respondents were not willing to accept worse levels of other attributes to avoid mild forms of these conditions (Figure A1). Following moderate-to-severe palmar-plantar syndrome and hypertension, respondents placed the most importance on an additional 10 months of maintaining daily function (from 3 months to 13 months) and an additional 10 months of OS (from 10 months to 20 months); however, differences between these estimates for maintaining daily function and additional months of OS were not statistically significant. Respondents placed the least importance on a 5-percentage-point reduction in the risk of bleeding in the digestive tract (from 7% to 2%), and this was not significantly different from the conditional relative importance of the dosing attribute (i.e., daily oral pill vs. intravenous [IV] infusion every 3 weeks). In evaluating attribute importance by key patient characteristics, we observed significant differences in patient preferences by transportation methods (see Appendix C). Significant differences were not observed between subgroups defined by age, race, geographic region, ability to perform daily activities, distance to cancer treatment, hypertension, or palmar-plantar syndrome.

### 3.3. Minimum Overall Survival Required to Offset Changes in Other Treatment Attributes

Five changes in attribute levels offered in the survey would have required more than 10 additional months of OS to offset, which is the largest increase in number of months of OS presented in the DCE experimental design (Table 3). These included a change in the number of months one is able to do activities without decline from 13 months to 3 months; a change in palmar-plantar syndrome from no or from mild palmar-plantar syndrome to moderate-to-severe palmar-plantar syndrome; and a change in hypertension from no or from mild hypertension to moderate-to-severe hypertension. The smallest MAB in terms of additional months of OS resulting from treatment was to accept a change in frequency of IV administration from every 4 weeks to every 3 weeks. On average, patients would only require an additional 0.39 months of OS to accept this change; however, this estimate is not statistically different from 0 at the 95% confidence level.

### 3.4. Predicted Choice Probabilities

**Figure 3:**
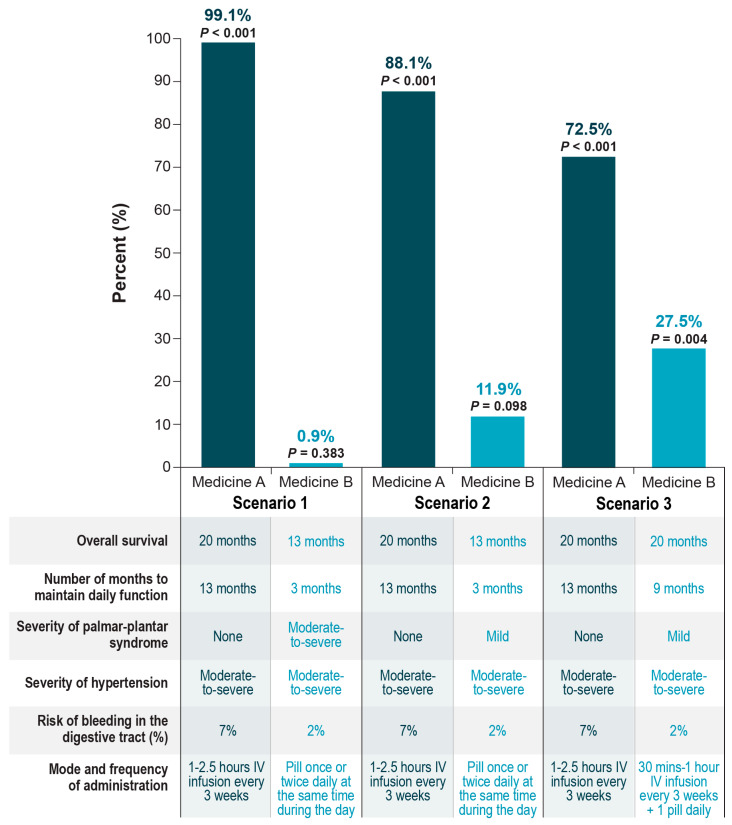
Respondent predicted choice probabilities. IV = intravenous.

In a simulation exercise to relate the preference weight estimates to scenarios representing real-world treatment choices, we estimated the probability that an average respondent would select one treatment profile over another, where the treatment profiles were defined by clinically plausible combinations of the attribute levels (Figure 3). Scenarios 1 and 2 were identical except for the level of palmar-plantar syndrome, which was moderate-to-severe for Medicine B in scenario 1 and was mild for Medicine B in scenario 2. Scenario 3 compared profiles for two medicines that differed in months of daily function, level of palmar-plantar syndrome, risk of bleeding in the digestive tract, and mode and frequency of administration. An average respondent had approximately a 99.1% likelihood of selecting Medicine A in scenario 1; approximately an 88.1% likelihood of selecting Medicine A in scenario 2; and approximately a 72.5% likelihood of selecting Medicine A in scenario 3. In scenario 1 and scenario 2, a greater risk of digestive tract bleeding in the digestive tract and IV dosing were outweighed by longer OS, maintaining daily function for more time, and a lack of palmar-plantar syndrome. In scenario 3, a greater risk of bleeding in the digestive tract was outweighed by a lack of palmar-plantar syndrome, maintaining daily function for more time, and IV-only dosing.

## 4. Discussion

This DCE study provides important insights into patient preferences for approved first-line HCC treatments in the US. Across treatment attributes included in the survey, patients prioritized avoiding severe AEs—including moderate-to-severe palmar-plantar syndrome and hypertension—that are known to negatively impact HRQOL. Differences in mode and frequency of treatment administration and avoiding a 2% to 7% risk of digestive tract bleeding were regarded as the least important treatment attributes among patients in our study. In simulations of patients’ choices between two hypothetical treatment profiles, patients were predicted to choose treatments that confer greater benefit in terms of OS and maintenance of daily function and to avoid moderate-to-severe palmar-plantar syndrome and hypertension.

Patients with unresectable HCC often face a poor prognosis and limited remaining life expectancy [21]. Therefore, treatment choices focus on maximizing survival benefits while maintaining HRQOL—which may be impaired by AEs common among HCC treatments, such as palmar-plantar syndrome, hypertension, and diarrhea. Despite the expansion of first-line systemic treatment options for unresectable HCC, the impact of various treatments on the HRQOL of patients has been variable. In the REFLECT trial, lenvatinib demonstrated noninferiority when compared to sorafenib in terms of OS, and patient-reported outcomes with lenvatinib were largely no different than with sorafenib in unresectable HCC patients [22]. While CheckMate 459 did not show a statistically significant improvement in OS with nivolumab versus sorafenib as first-line treatment in patients with unresectable HCC, significant improvements in total, physical, and functional well-being scores using the Functional Assessment of Cancer Therapy Hepatobiliary Cancer questionnaire were reported with nivolumab compared to sorafenib [23]. In contrast, the IMbrave150 study demonstrated that atezolizumab plus bevacizumab provided both statistically significant improvements in OS and progression-free survival and clinically meaningful benefits in patient-reported HRQOL, functioning, and disease symptoms versus sorafenib for patients undergoing first-line treatment for HCC, despite an increase in bleeding risk [8].

Patients in this study required more than 10 months of additional OS to compensate for moderate-to-severe palmar-plantar syndrome or hypertension, while requiring only about 1.6 months of additional OS to compensate for an increase in the risk of gastrointestinal bleeding from 2% to 7% and approximately 0.39 months of additional OS to accept a change in IV infusion frequency from every 4 weeks to every 3 weeks. In addition, on average, patients generally did not require statistically significant increases in efficacy, defined as additional months of OS, to accept moderate increases in risk of bleeding or IV administration. The degree to which respondents prioritized avoiding moderate-to-severe palmar-plantar syndrome and moderate-to-severe hypertension is somewhat challenging to interpret. In our survey, moderate-to-severe palmar-plantar syndrome was described as potentially impacting the ability to conduct instrumental and self-care activities of daily living, while moderate-to-severe hypertension included the description of serious risks and side effects associated with hypertension (e.g., risk of seizure, stroke, or heart attack). Respondents’ hypothetical treatment choices may have been influenced by the more severe aspects of these attributes, as informed by the descriptions used in the survey. Interestingly, a previous DCE in HCC similarly revealed respondents to be strongly averse to severe hypertension; these respondents were willing to forgo 1.6 months of additional OS to reduce the risk of hypertension by 10% [10]. While these results cannot be directly compared with our findings because the studies used different sets of experimentally designed attributes and levels, both demonstrate that patients will forgo survival gains to avoid serious AEs, suggesting the prioritization of HRQOL above survival for some patients with advanced HCC.

Patient preferences regarding efficacy, toxicity, mode of administration, and the HRQOL impact of systemic therapies in HCC are increasingly important to recognize but have been critically understudied as new treatment options for unresectable HCC become available. These results may inform shared decision-making discussions between physicians and patients to guide the selection of treatments that best fits patients’ priorities. To our knowledge, our study is the first to elicit patients’ preferences for HRQOL as reflected by the ability to conduct daily activities. Our findings show that, from the patient perspective, the ability to maintain quality of life and conduct daily activities while undergoing first-line treatment for HCC is as important or more important than a treatment’s survival benefit. In order to address this important patient perspective, the regulatory approval of future therapies in HCC should be based not only on survival benefit but also on the ability to improve HRQOL for patients receiving treatment. Given the evolving paradigm for first-line treatment of HCC, future research may focus on further exploring patient preferences for various different CIT-based regimens.

The limitations of this study, including some inherent to DCE surveys, must be considered. The data collected in DCEs are based on responses to hypothetical choice profiles. Attempts were made to limit potential hypothetical bias by constructing choice questions that mimic realistic clinical choices as closely as possible and map clearly onto clinical evidence. However, DCE surveys can accommodate only a limited number of treatment attributes, so the study only includes a subset of the outcomes and burdens associated with HCC treatment. In particular, some immune-mediated AEs that may be relevant to patients were not included in the DCE. Detailed information on respondents’ experiences with therapy, including specific regimens, was not collected. The respondent sample may also not reflect the US population with HCC, potentially limiting representativeness. The survey was written in English only, and the results might only be representative of US patients whose primary language is English. Finally, we report mean relative preference estimates for each attribute level from the RPL model, which may mask heterogeneity in preferences across the sample. The subgroup results suggest that there is heterogeneity in preferences, at least for some patient characteristics.

## 5. Conclusions

Patients with unresectable HCC place much more importance on avoiding side effects that would severely impact their HRQOL during treatment than on the mode and frequency of treatment administration and the risk of digestive tract bleeding. Some patients with unresectable HCC prioritize the ability to maintain HRQOL and to conduct daily activities as much as or more than they do a treatment’s survival benefit. The results of this study provide insight into patient preferences across attributes of treatment for unresectable HCC and may help physicians to select treatments that best fit patients’ preferences and support shared decision-making.

## Figures and Tables

**Table 1:** Attributes and levels for the discrete-choice experiment survey.

Type of Attribute	Patient-Friendly Attribute Label	Possible Attribute Levels
Benefit	How long can the medicine extend your life	20 months 13 months 10 months
Benefit	Number of months to noticeable decline in ability to do activities	13 months 9 months 3 months
Adverse event	Redness and sores on your hands and feet	None Mild Moderate-to-severe
Adverse event	High blood pressure	None Mild Moderate-to-severe
Risk of adverse event	Risk of bleeding in the digestive tract	2 people out of 100 (2%) 5 people out of 100 (5%) 7 people out of 100 (7%)
Process	How you take the medicine, how often and where	Pill once or twice daily at the same time during the day 1 to 2½ h IV infusion every 3 weeks 1 to 2½ h IV infusion every 4 weeks 30 min to 1 h IV infusion every 3 weeks + a pill daily

IV = intravenous infusion. Note: attribute descriptions from the survey are presented in Appendix A (Table A1).

**Table 2:** Summary of patient characteristics.

	*N* = 200
Age (mean), years (SD)	58.9 (11.2)
Male	111 (55.5%)
Race or ethnicity, ^a^ n (%)	
Black or African American	57 (28.5%)
Hispanic or Latino	29 (14.5%)
White	42 (21.0%)
A race not listed or prefer not to answer ^b^	72 (36.0%)
Education level, n (%)	
High school degree or equivalent	43 (21.5%)
Some college but no degree, trade/technical/vocational school, 2-year college degree, or other	74 (37.0%)
4-year college degree or greater	83 (41.5%)
Pre-tax household income in 2020, n (%)	
Less than USD 50,000	67 (33.5%)
USD 50,000 to USD 99,999	60 (30.0%)
USD 100,000 to USD 149,999	12 (6.0%)
USD 150,000 or more	43 (21.5%)
Do not know/not sure or prefer not to say	18 (9.0%)
Please select the response that you think best describes how you feel currently, n (%)	
I need some help taking care of myself, and I spend more than half of waking hours in a bed or chair	46 (23.0%)
Time since diagnosis, n (%)	
Less than 1 year ago	72 (36.0%)
1 year to fewer than 5 years ago	114 (57.0%)
More than 5 years ago	14 (7.0%)
Currently receiving treatment for HCC	171 (85.5%)
Among those currently receiving treatment for HCC: ^a^	*n* = 171
Medicines via IV	88 (51.5%)
Medicines as oral pills	122 (71.3%)
All respondents	
Has your doctor ever diagnosed you with high blood pressure?	
Yes	52 (26.0%)
No	146 (73.0%)
Don’t know or not sure	2 (1.0%)
Among those who have been diagnosed with high blood pressure:	*n* = 52
Using the following definitions, how would you describe your high blood pressure?	
Mild ^c^	31 (59.6%)
Moderate-to-severe ^d^	21 (40.4%)
Don’t know or not sure	0 (0.0%)
Among those who are currently receiving or previously received treatment for HCC:	*n* = 186
Have you ever experienced redness and sores on your hands and feet as a side effect of your liver cancer treatment?	
Yes	82 (44.1%)
No	102 (54.8%)
Don’t know or not sure	2 (1.1%)
Among those who have experienced redness and sores as a side effect of treatment for HCC:	*n* = 82
Using the following definitions, how would you describe the redness and sores on your hands and feet you experienced?	
Mild ^e^	48 (58.5%)
Moderate-to-severe ^f^	34 (41.5%)
Don’t know or not sure	0 (0.0%)

HCC = hepatocellular carcinoma; IV = intravenous; SD = standard deviation. ^a^ Respondents could select more than 1 response option, so percentages may not total 100%. ^b^ This category also included American Indian or Alaska Native, Middle Eastern or North African, and Native Hawaiian or other Pacific Islander. ^c^ Mild high blood pressure was defined as follows: Your blood pressure increases, but it can be managed with medication. You do not experience any symptoms, except side effects from blood pressure medicine. ^d^ Moderate-to-severe high blood pressure was defined as follows: If you develop moderate-to-severe high blood pressure, you will need to take additional medicine and your doctor will need to monitor your blood pressure more frequently. People with moderate-to-severe high blood pressure can experience moderate-to-severe chest pain, headache, confusion and blurred vision, nausea and vomiting, and shortness of breath. Moderate-to-severe high blood pressure puts you at risk of having seizures, a stroke or heart attack. ^e^ Mild redness and sores on the hands and feet were described as follows: You have mild dryness or swelling on the palms of your hands and/or on the soles of your feet, but it will not be painful. You can do your normal activities. ^f^ Moderate-to-severe redness and sores on the hands and feet were described as follows: You have moderate to severe pain, dryness, peeling, bleeding, or blistering on the palms of your hands and/or on the soles of your feet. The pain may make it difficult to walk or use your hands and/or feet. You have some trouble with moderate physical activities such as walking, housework, and shopping. You have some trouble doing your normal work and social activities. If the symptoms are severe, you need help taking care of yourself including bathing, dressing, feeding yourself, using the toilet, and taking medications.

**Table 3:** Minimum acceptable benefit as an increase in additional months of overall survival for a given change in treatment attributes.

Attributes	From Level	To Level	MAB (*N* = 200)	95% CI	
Number of months you are able to do activities without decline	13 months	3 months	>10	N/A	
	13 months	9 months	2.31	0.56	4.06
	9 months	3 months	4.69	−2.66	12.03
Redness and sores on your hands and feet	None	Moderate-to-severe	>10	N/A	
	None	Mild	0.87	−0.30	2.01
	Mild	Moderate-to-severe	>10	N/A	
High blood pressure	None	Moderate-to-severe	>10	N/A	
	None	Mild	1.77	0.40	3.14
	Mild	Moderate-to-severe	> 10	N/A	
Risk of bleeding in the digestive tract	2 people out of 100 (2%)	7 people out of 100 (7%)	1.60	0.04	3.17
	2 people out of 100 (2%)	5 people out of 100 (5%)	0.43	−0.73	1.59
	5 people out of 100 (5%)	7 people out of 100 (7%)	1.17	−0.06	2.40
How you take the medicine, how often and where	Pill once or twice daily at the same time during the day	30 min to 1 h IV infusion every 3 weeks + a pill daily	2.21	0.57	3.85
	Pill once or twice daily at the same time during the day	1 to 2½ h IV infusion every 4 weeks	1.29	−0.19	2.76
	Pill once or twice daily at the same time during the day	1 to 2½ h IV infusion every 3 weeks	0.89	−0.55	2.34
	1 to 2½ h IV infusion every 3 weeks	30 min to 1 h IV infusion every 3 weeks + a pill daily	1.32	−0.13	2.76
	1 to 2½ h IV infusion every 4 weeks	1 to 2½ h IV infusion every 3 weeks	0.39	−0.93	1.72
	1 to 2½ h IV infusion every 4 weeks	30 min to 1 h IV infusion every 3 weeks + a pill daily	0.92	−0.44	2.28

CI = confidence interval; IV = intravenous; MAB = minimum acceptable benefit; N/A = not available; OS = overall survival. Note: When the 95% CI around a mean MAB includes 0, the mean MAB is not statistically different from 0. In this study, MABs are computed in terms of increases in months of OS (over a baseline of 10 months) resulting from treatment. These calculations hold constant the levels of all attributes other than the one attribute that is changing. Instead of making the strong assumption that the utility of each additional month of OS remained constant beyond 10 months of increase (which was the largest difference in additional months of OS presented in the study), MAB estimates greater than 10 months of additional OS were reported as “>10,” and CIs were not reported.

## Data Availability

The study sponsor will make the data available to other researchers upon reasonable request.

## Appendix A. Attribute Descriptions from the Survey

**Table A1:** Attribute Descriptions.

Patient-Friendly Attribute Label	Description in Survey
How long can the medicine extend your life	An important goal of cancer medicines is to increase the length of time during and after the treatment that a patient lives with the cancer. Different medicines work for different amounts of time. We will ask you to think about medicines that can extend your life between 10 months and 20 months.
Number of months to noticeable decline in ability to do activities	Some cancer medicines can help maintain your ability to perform daily activities without noticeable difficulty while you are taking the medicine. For example, you can continue to do things like take a walk, carry shopping bags, and take care of yourself (eating, dressing, and washing up) without additional difficulty while on treatment. We will ask you to think about medicines that allow you to keep doing your activities with very little difficulty for 3 months to 13 months. After this time, your ability to do these activities will noticeably decline.
Redness and sores on your hands and feet	Some cancer medicines increase the risk of a rash that causes redness, swelling, blisters, and pain on your hands or feet. We will ask you to think about cancer medicines that may result in different levels of rash that causes redness, swelling, blisters, and pain. There are three possibilities. None: ▪ You will not experience any problems with your hands or feet. Mild: ▪ You have mild dryness or swelling on the palms of your hands and/or on the soles of your feet, but it will not be painful. ▪ You can do your normal activities. Moderate-to-severe ▪ You have moderate to severe pain, dryness, peeling, bleeding, or blistering on the palms of your hands and/or on the soles of your feet. ▪ The pain may make it difficult to walk or use your hands and/or feet. ▪ You have some trouble with moderate physical activities such as walking, housework, and shopping. You have some trouble doing your normal work and social activities. ▪ If the symptoms are severe, you need help taking care of yourself including bathing, dressing, feeding yourself, using the toilet, and taking medications.
High blood pressure	Some cancer medicines can cause an increase in blood pressure. People who already have high blood pressure may have an increase in their blood pressure. People who do not have high blood pressure may develop it. Generally, there are no symptoms associated with high blood pressure, but high blood pressure can lead to more serious health problems and needs to be monitored and controlled as part of the disease. Untreated high blood pressure increases your risk of serious health problems such as severe headache, heart attack, stroke, or kidney disease. Your doctor will check your blood pressure every month. If your blood pressure increases while taking cancer medicines, your doctor may start you on blood pressure medicines or change your current blood pressure medicines to lower your blood pressure to the normal range. Side effects of blood pressure medicines can include dizziness, drowsiness, headaches, weakness, dry mouth, or other side effects. One in 10 patients will have one of these side effects from the high blood pressure medicines. We will ask you to think about cancer medicines that may result in different levels of high blood pressure. There are three possibilities. None: ▪ The medicine does not affect your blood pressure. Mild high blood pressure: ▪ Your blood pressure increases, but it can be managed with medication. You do not experience any symptoms, except side effects from blood pressure medicine. Moderate-to-severe high blood pressure: ▪ If you develop moderate-to-severe high blood pressure, you will need to take additional medicine and your doctor will need to monitor your blood pressure more frequently. ▪ People with moderate-to-severe high blood pressure can experience moderate-to-severe chest pain, headache, confusion and blurred vision, nausea and vomiting, and shortness of breath. ▪ Moderate-to-severe high blood pressure puts you at risk of having seizures, a stroke or heart attack.
Risk of bleeding in the digestive tract	Patients with liver cancer can experience bleeding in their digestive tract as a symptom of the cancer. Some medicines may also cause bleeding in the digestive tract during treatment. When patients take a medicine that may cause digestive tract bleeding, the doctor may do tests to check whether the patient is at risk of bleeding. If the doctor decides the patient is not at risk of bleeding, most patients can take the medicine and have no bleeding problems. However, even after testing, some patients who take the medicine will develop bleeding in the digestive tract. People with gastrointestinal (GI) bleeding can experience severe problems like anemia, or even shock, and they may need to be treated at the hospital. Gastrointestinal bleeding can be life-threatening if it is not treated. Your doctor will monitor you for signs of GI bleeding after you start treatment. Later in this survey, we will ask you to think about cancer medicines that have different risks of GI bleeding that range from a 2% risk to a 7% risk.
How you take the medicine, how often and where	Medicines used to treat cancer can be taken as a pill or by an intravenous (IV) infusion, and they can be taken on different schedules. They can also be taken at home or in a doctor’s office, outpatient clinic, or hospital. ▪ If you take the medicine in a doctor’s office, outpatient clinic, or hospital, nurses will monitor you for side effects and they can take blood for tests you might need. ▪ If you take the medicine at home, you can monitor yourself and call your doctor’s office if you have questions. You will need to visit the doctor’s office for any additional tests. To get an IV infusion, a nurse will insert a needle into a vein in the patient’s arm that is attached to a bag filled with liquid medicine. The patient will sit for about 30 min to 2.5 h while the medicine goes into their vein. We will ask you to think about medicines that can be taken in different ways and on different schedules. There are four possibilities: Pill once or twice daily at the same time during the day: ▪ Every day, the patient takes a pill with water at home once or twice a day at the same time during the day 1 to 2½ h IV infusion every 3 weeks: ▪ Every 3 weeks, the patient gets an IV infusion at their doctor’s office, hospital, or an outpatient clinic ▪ The infusion will take 1 to 2½ h 1 to 2½ h IV infusion every 4 weeks: ▪ Every 4 weeks, the patient gets an IV infusion at their doctor’s office, hospital, or an outpatient clinic ▪ The infusion will take 1 to 2½ h 30 min to 1 h IV infusion every 3 weeks + a pill daily: ▪ Every day, the patient takes a pill with water at home once or twice a day at the same time during the day ▪ Every 3 weeks, the patient also gets an IV infusion at their doctor’s office, hospital, or an outpatient clinic ▪ The infusion will take 30 min to 1 h

## Appendix B. Preference Weight Analysis

Preferences for the full sample were ordered as expected, with better levels being preferred to worse levels. Overall, respondents preferred the following:

- Treatment that extends life for longer
- Treatment that provides more months in which respondents are able to do activities without decline
- Treatment that causes less-severe palmar-plantar syndrome (although respondents were indifferent to changes in palmar-plantar syndrome from mild to none)
- Treatment that causes less-severe hypertension
- Treatment with less risk of bleeding in the digestive tract (although respondents were indifferent to the risk of bleeding in the digestive tract from treatment decreasing from 5% to 2% and from 7% to 5%)
- Treatment that can be orally administered during the day compared with 30 min to 1 h IV infusion every 3 weeks plus a pill daily (although respondents were indifferent to changes across the other levels in mode and frequency of administration)

All attribute levels for additional months of OS resulting from treatment, time until decline in ability to perform activities, and severity of high blood pressure were statistically different from each other within the attribute. A Wald χ^2^ test was used to determine the statistical significance of differences between adjacent attribute levels. Statistical significance was determined if *p* < 0.05.

Figure A1 presents the normalized mean preference weight estimates for each attribute level for the full sample. The change in utility associated with a change from 1 level of an attribute to another for each attribute is represented by the difference between the preference weights between the levels. Larger differences between preference weights indicate that respondents viewed the change as having a relatively greater effect on overall utility.

## Appendix C. Subgroup Analysis

We tested for differences in preferences by 9 sets of self-reported characteristics. Table A2 presents the 9 subgroups tested with the *p* values from the log likelihood χ^2^ test of joint statistical significance of the interaction terms for the subgroup. Subgroups with statistically different preferences (*p* < 0.05) were analyzed further. Only 2 subgroups of the 9 total tested subgroups were statistically significant:

- Self-transportation: respondents who walk or drive themselves to their cancer treatments at least some of the time versus respondents who never walk or drive themselves to their cancer treatments
- Public transportation: respondents who use public or hospital transportation to get to their cancer treatments versus respondents who never use public or hospital transportation to get to their cancer treatments

**Table A2:** Descriptions of the Subgroups Analyzed (*N* = 200).

Subgroup Pair	Subgroup Description	Sample Size (*N* = 200)	*p* Value from the Log Likelihood $\chi^{2}$ Test of Joint Statistical Significance
Aged 70 years or older	Younger than 70 years	172	0.491
	70 years or older	28	
Race	Does not identify as White ^a^	126	0.733
	Exclusively White	42	
Geographic region	Lives in the Midwest, South, or Northeast	75	0.359
	Lives in the West	125	
Ability	Is unable to do work activities or needs help taking care of self	98	0.629
	Is fully active or is able to do activities that are not physically demanding	102	
Distance to cancer treatment	Travels less than 30 min to cancer treatments	62	0.838
	Travels 30 min or more to cancer treatments	137	
Self-transportation	Never walks or drives themselves to cancer treatments	116	0.018
	Walks or drives themselves to cancer treatments at least some of the time	84	
Public/hospital transportation	Never uses public or hospital transportation to cancer treatments	142	0.009
	Uses public or hospital transportation to cancer treatments at least some of the time	58	
Palmar-plantar syndrome	Has never experienced palmar-plantar syndrome as a side effect of treatment for HCC	102	0.374
	Has experienced palmar-plantar syndrome as a side effect of treatment for HCC	82	
Hypertension	Has never been diagnosed with hypertension	146	0.554
	Has been diagnosed with hypertension	52	

^a^ “Does not identify as White” comprises respondents who identify as American Indian or Alaska Native, Asian, Black or African American, Hispanic or Latino, Middle Eastern or North African, Native Hawaiian or other Pacific Islander, and/or a race not listed. Note: Frequencies across subgroup pairs may not add to 200 due to missing values.
